# Mediating effects of social support, mental health between stress overload, fatigue and turnover intention among operating theatre nurses

**DOI:** 10.1186/s12912-023-01518-z

**Published:** 2023-10-06

**Authors:** Jia-Bin Xu, Qing-Xiang Zheng, Xiu-Min Jiang, Qing Zhuo, Jin-Xia Nian, Jie-Ting Wang

**Affiliations:** 1 Nursing Department, Fujian Maternity and Child Health Hospital College of Clinical Medicine for Obstetrics & Gynecology and Pediatrics, Fujian Medical University, No.18 Daoshan Street, Gulou District, Fuzhou City, Fujian Province China; 2 Fujian Branch of Shanghai Children’s Medical Center, Shanghai Jiaotong University school of Medicine, Fuzhou City, Fujian Province China; 3 Fujian Children’s Hospital, Fuzhou City, Fujian Province China; 4 Fujian Obstetrics and Gynecology Hospital, Fuzhou City, Fujian Province China

**Keywords:** Operating theatre nurses, Turnover intention, Fatigue, Social support, Stress overload, Structural equation model

## Abstract

**Background:**

The high rate of nurses turnover and nursing staff shortage have been an ongoing concern issue and a challenge for global health systems. To explore the turnover intention among operating theatre nurses, and to test the hypothetical model for estimating the effects of stress overload and fatigue between social support, mental health and turnover intention.

**Design:**

a multi-center and cross-sectional online survey.

**Methods:**

This study was conducted from October 2020 to March 2021 comprised 1060 operating theatre nurses from 76 Chinese hospitals. The descriptive analysis, independent sample *t* test and one-way analysis of variance and Spearman correlation analysis were used to explore the relationships among variables by the SPSS software, and stepwise multiple linear regression analysis was utilized to identify influencing factors of turnover intention and its dimensions among operating theatre nurses. A structural equation model was analyzed by the AMOS software.

**Results:**

Social support, mental health, stress overload and fatigue were important predictors of turnover intention among operating theatre nurses. Besides, stress overload positively affected fatigue, mental health and turnover intention; fatigue negatively affected social support, however, fatigue positively affected mental health; social support negatively affected mental health and turnover intention; mental health positively affected turnover intention. Moreover, social support, mental health mediated between stress overload, fatigue and turnover intention among operating theatre nurses.

**Conclusion:**

Social support, mental health mediated between stress overload, fatigue and turnover intention among operating theatre nurses.

## Introduction

Nurses turnover intention is not only a predictor before the occurrence of turnover behavior, but also affect their work enthusiasm and stability, and the nursing service quality [1]. The high rate of nurses turnover and nursing staff shortage have been an ongoing concern issue and a challenge for global health systems [2, 3]. There was a global shortage of nearly 6 million nurses [4]. One of the important causes of the nursing staff shortage is the high nurses turnover rate [5, 6]. If the reasons for nurses turnover intention are carefully analyzed and discussed by nursing managers, and then they take timely intervention measures to deal with the factors of nurses turnover intention, it may decrease the occurrence of turnover behavior, even attract the lost personnel back to the original nursing position [7]. So it is necessary for nursing managers to carefully explore the factors of nurses turnover intention from the perspective of in-service nurses.

It well known that the operating theatre nurses are required to be responsible for asepsis, instrumentation, cooperating in a surgical team, infection and complication control, and biological specimens management during the surgical procedure [8, 9]. Besides, they also have to cater to different personalities and surgical techniques of different surgeons with one- to five-year developmental cycle [10]. So compared with nurses from other nursing care units, operating theatre nurses need to be trained with more time, energy and money [11]. Moreover, the fast-paced and high-technology instruments and surgical medical techniques in operating theatre offer various challenges for operating theatre nurses, they need to keep lifelong learning of new skills. So it is very difficult for nursing managers to recruit and retain operating theatre nurses [12, 13]. The turnover of operating theatre nurses might further worsen the nurses shortage. Therefore, exploring turnover intention of operating theatre nurses in-service is vital to prevent them turnover, and retain them in this unique and challenging clinical environment.

The turnover of specialized nurses in ICU, emergency department and pediatrics has been widely concerned by researchers and nursing managers, however, there are few studies which concentrate on the turnover or turnover intention of operating theatre nurses. Chen found that there were 78.64% (162 of 206) operating theatre nurses having a moderate and high turnover intention, and the influencing factors of turnover intention included salary, sense of fairness, operating theatre work characteristics, age, promotion opportunities and management methods [14]. Besides, the COVID-19 pandemic had caused the increase of nurses turnover intention rate [15–17]. Because the COVID-19 pandemic had dramatically increased the nursing workforce [18, 19]. Meanwhile, nurses might have strong emotional reactions to the COVID-19 pandemic, including fear, anger, frustration and worries, which could hinder their professional performance [18]. And sustained exposure to COVID-19 patients and work overload had caused health staff stress, fatigue, anxiety and other detriments [20]. In particular, being faced with the high risk of exposure to COVID-19 patients, operating theatre nurses and anesthesiologists had high incidence of anxiety and depression [21]. However, few studies focus on the relationships among turnover intention, stress overload, fatigue, mental health and social support.

Stress overload is a new approach to estimate stress [22]. Stress overload refers to the maximum stress state that it can accommodate, paying more attention to the psychological stress [23]. Job stress was prevalent in nursing positions [24], due to high work intensity, irregular rest state of the night shift, the accusations from the patients and their families, too many hospital exams, fewer opportunities for promotion, and tension relationship with colleagues [23]. Nurses not only play an important roles of caregivers and educators in the hospitals, but also play roles of mother or child in their family. The multiplicity and particularity of above roles determine that their stress overload is much higher than that in other occupations [23]. Meanwhile, the stress overload among emergency department nurses were positively related with turnover intention [23]. In addition, Yang et al. found that nurses challenge stress was inversely associated with turnover intention, while hindrance stress was positively correlated with their turnover intention [25]. However, the relationship between stress overload and turnover intention among operating theatre nurses is not clear. Besides, job stress was highly associated with fatigue and depression [24].

Fatigue is defined as a decline in the body’s autonomous activity with non-specific symptom due to long-term body overwork, prolonged emotional stress, or regular lack of sleep [26, 27]. 83% of nurses said that their medical errors were due to fatigue [28]. Several studies showed that burnout was a key factor in nurses’ intention to leave their job and/or the nursing profession [7, 29]; and burnout was also positively associated with nurses turnover intention [6, 7, 16, 30]. In fact, fatigue is a main feature of the burnout syndrome [31]. Fatigue means that extreme tiredness resulting from mental or physical exertion or illness, while burnout means that a state of emotional, physical, and mental exhaustion caused by excessive and prolonged stress [32]. And there are few studies to explore the fatigue and turnover intention among operating theatre nurses.

Mental health refers to the psychological aspects and activities in a normal and good positive state. The nurses’ mental health was directly related to patient individual well-being and safety outcomes [33, 34]. More social support and social recognition for operating theatre nurses might potentially help them relieve their psychological pressure [34]. However, nurses are often vulnerable to mental health challenges when exposed to high workload everyday [34]. Furthermore, unfavorable mental health conditions among nurses were positively correlated with high rate of nurses turnover intention, such as depression and anxiety [34].

Social support refer to the help from individuals families, friends, colleagues and other important people when individuals are under pressure, which can help individuals to better deal with difficulties and crises encountered in their life [35]. Low level of social support may increase the level of nurses burnout [36]. Besides, social support was negatively correlated with nurses turnover intention [35]. However, Li et al. found that social support was not clearly associated with turnover intention among 1,313 newly graduated nurses [21]. And whether social support is associated with turnover intention among operating theatre nurses that is also not clear. Therefore, it is significant to further the relationship between social support and fatigue/turnover intention among operating theatre nurses.

Based on above references, few studies examine the relationships among turnover intention, stress overload, fatigue, mental health and social support among operating theatre nurses. In this study, we aimed to explore the turnover intention among operating theatre nurses, and to test the hypothetical model for estimating the effects of stress overload and fatigue between social support, mental health and turnover intention.

### A hypothetical model

**Fig. 1:**
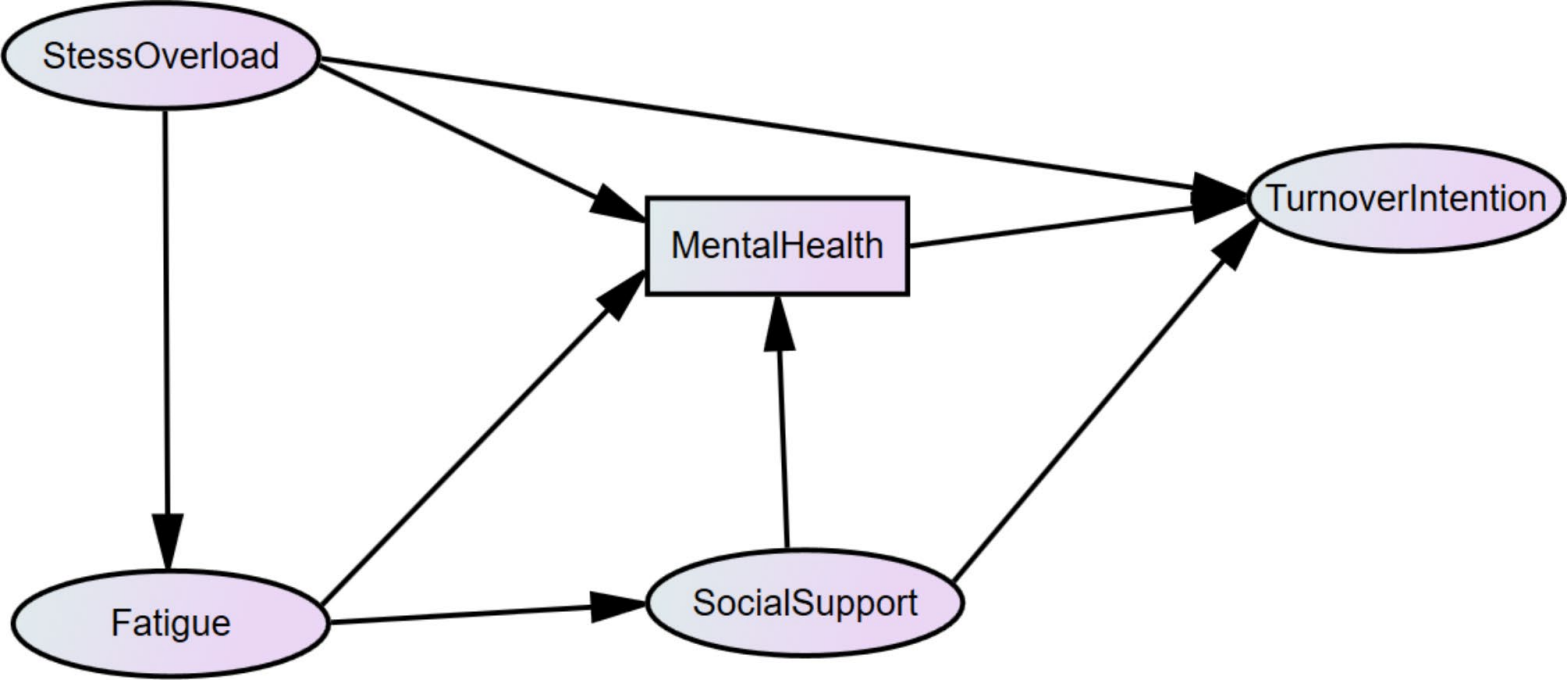
Theoretical model and hypotheses

Based on the literature review, the preassumption of model was presented in Fig. 1. Meanwhile, the hypothesis for that was as follows: Hypothesis 1: Stress overload has a significant direct effect on fatigue. Hypothesis 2: Stress overload has a significant direct and total effects on mental health. Hypothesis 3: Stress overload has a significant direct and total effect on turnover intention, and stress overload had significant indirect effects on turnover intention via fatigue and social support. Hypothesis 4: Fatigue had a significant direct effect on social support. Hypothesis 5: Fatigue had a significant direct and total effect on mental health. Hypothesis 6: Fatigue had significant indirect effects on mental health via social support. Hypothesis 7: Social support had a significant direct effect on mental health. Hypothesis 8: Social support had a significant direct and total effects on turnover intention, and fatigue had significant indirect effects on turnover intention via mental health.

## Methods

### Aims

The study aims to explore the turnover intention among operating theatre nurses, and to test the hypothetical model for estimating the effects of stress overload and fatigue between social support, mental health and turnover intention.

### Study design and participants

A multi-center, cross-sectional survey design was used to test our hypothesized model. This survey were involved totally 1100 operating theatre nurses by convenient sampling method, and they came from 76 Chinese hospitals including 60 general hospitals and 16 special hospitals. The inclusion criteria were as follows: (a) registered nurses; (b) age ≥ 18 years old; (c) operating theatre nurses were responsible for clinical work at operating theatre; (d) nurses were without mental or cognitive impairment; (e) nurses voluntarily participated in this study. The exclusion criteria included: (a) nurses engaged in advanced studies in other hospital or had a leave during investigation; (b) nurses were in continuing study for master’s degree or above; (c) nurses were in internship time or probation period.

### Data collection

This study was conducted from October 2020 to March 2021. This study distributed questionnaires by online questionnaire system via WenJuanXing a professional online questionnaire platform (https://www.wjx.cn/). Firstly, the questionnaires of this survey were prepared on WenJuanXing platform, and then a quick respond (QR) code of the questionnaires was formed from this platform. Secondly, informed consents were got from the nursing manager of each hospital, and then the QR code was sent to the head nurses of operating theatre who helped to sent the QR code to each operating theatre nurse. Thirdly, operating theatre nurses could get access to use WeChat to sweep the QR code and then fill in the questionnaires. Especially, operating theatre nurses participated in this survey by the principle of voluntariness, and they had right to reject this participation. Lastly, the researcher collected data from WenJuanXing platform. In order to keep data accuracy, the requirement was set that each question of the questionnaires must be answered, and each participant had only one chance to complete all questions, otherwise it could not be submitted.

### Questionnaires

The study data was collected via WenJuanXing in China. The online questionnaires of this study were composed of six parts, including a general information questionnaire, turnover intention questionnaire, the Chinese version of Percieved Social Support questionnaire, the General Health Questionnaire-12 questionnaire, the Chinese version of Stress Overload questionnaire and the Chinese version of Chalder Fatigue Scale.

### General questionnaire

A general questionnaire was designed by the researchers to collect the social-demographic and working characteristics of operating theatre nurses. The social-demographic characteristics composed of gender, age, education level, marital status, fertility status, situation of raising children, living status, times and ways of weekly exercise, having chronic disease or not and suffering negative life events or not. The working characteristics included working experiences in nursing, working experience in operating theatre, monthly income, weekly working hours, times of monthly night shifts and nursing professional title.

### Turnover intention

The turnover intention questionnaire, also named as Intention to Leave questionnaire, contained three dimensions: intention to leave unit, intention to leave organization and intention to leave profession [37]. And this questionnaire have six items. The intention to leave unit is measured one item, the intention to leave organization is measured by four items and the intention to leave profession is measured by one item. Each item is scored by 5-point Likert scale method [37, 38]. The total score is a total of each item score. Higher scores indicate higher intention to leave. The turnover intention questionnaire with a good internal consistency were widely used in the nurses [6, 37, 38]. The Cronbach’s α value in this questionnaire was 0.943.

### Stress overload

The stress overload scale is developed by Amirkhan [22]. And this questionnaire is translated and revised by Chinese researcher due to culture adaptation [39]. It has been proved that the Chinese version of stress overload scale has good reliability and validity. The scale has two dimensions with 22 items, including event load (12 items) and individual vulnerability (10 items). Each item is scored by 5-point Likert scale method. The total score ranges from 22 to 110. The higher the score is, the greater the stress overload will be. The Cronbach’s α value for this scale and its dimensions in previous study were more than 0.85, which suggested this scale had a good internal consistency [23]. This previous study explored stress overload and turnover intention among emergency department nurses [23]. The Cronbach’s α value for this scale was 0.980 in this study.

### Fatigue

The fatigue scale is developed to assess individual’s fatigue severity by Chalder et al. [40], which consists of 14 items and two dimensions, including physical fatigue (item 1 to item 8) and psychological fatigue (item 9 to item 14) [40]. The Chinese version of fatigue scale, named as FS-14, is translated from the original Chalder’s fatigue scale. The total score of the fatigue scale is the sum of all items, and each item is scored by 2-point Likert scale method (1 = presence, 0 = absence). The total fatigue score ranges from 0 to 14. It has been shown that when the score is equal to 7 or more, the fatigue occurs [40]. The fatigue scale has been shown a satisfactory validity [41]. The scale had good reliability and validity with Cronbach’s α coefficient of 0.809 and half reliability of 0.676. The Cronbach’s α value for this scale in this study was 0.815.

### The 12-item general health questionnaire

The 12-item general health questionnaire (GHQ-12) is originally designed to assess individual’s mental health status over the past month [42]. It is composed of 12 items, and each item is evaluated by four indexes named as “never”, “rarely”, “sometimes” and “often”. A total of 12 items are divided into positive statement and negative statement. Each item has a score of 0 or 1, and the total score ranges from 0 to 12. The lower the score, the less the possibility of psychological disorders. Regarding to the optimal cut-off value of GHQ-12, the subjects were divided into two groups: high risk situation (GHQ-12 score ≥ 4 points) and low risk situation (GHQ-12 score < 4 points). The Chinese version of the GHQ-12 have been demonstrated by an amount of studies as a assessment tool having satisfactory reliability and sensitivity [43, 44]. The Cronbach’s alpha value of the GHQ-12 was 0.844 [43]. The Cronbach’s α value for this questionnaire in this study was 0.670.

### Perceived social support

The Perceived social support is firstly developed by Zimet et al. to assess individuals perceived social support which contain family, friends and other support dimensions and 12 items [45]. Each item is rated by 7-point Likert scale method, ranging from 1 to 7. The total scores range from 12 to 84, and higher scores indicate higher levels of perceived social support. The Cronbach’s α coefficient of the original scale was 0.880, and the Cronbach’s α value in this study was 0.978.

### Ethical considerations

This study received the ethics approval from the Ethics Committee of Fujian Maternity and Child Health Hospital (No. 2020YJ234). Besides, this study was also performed in accordance with ethical procedures, and all ethical consideration processes followed by the researcher aimed to protect participants. All data would be research data, and any participant could not be identified.

### Data analysis

The study data were analyzed by the SPSS (26.0 version) and the AMOS (25.0 version). Descriptive analysis, independent sample *t* test and one-way analysis of variance (ANOVA), were used to analyze the turnover intention, social support, stress overload and fatigue. The Spearman correlation analysis method was used to determine the correlation strength and direction between variables. Multiple linear regression analysis was utilized to identify influencing factors of turnover intention among operating theatre nurses. And all independent variables were entered in the multiple linear regression models. After above analyses, a structural equation model was conducted to explore the effects of stress overload and fatigue between social support, mental health and turnover intention by AMOS 26.0. The *p*-value below 0.05 was considered statistically significant.

To evaluate the fitness of the hypothetical model, the value of the χ^2^/df is recommended being below 3 [46]. Especially, the smaller χ^2^/df, the higher fit of the model. When χ^2^/df is below 3 indicates that the model is in the ideal state, and χ^2^/df is below 5 indicates that the model is also acceptable. Moreover, RMSEA is below 0.06 that indicate a good model fit [47]. Besides, the GFI and AGFI are great than 0.80, the IFI and CFI are great than 0.90 [46]. A maximum likelihood estimation method for covariance matrices were performed in this study. The indirect and total effects of the model were tested by Bootstrapping (5000 times).

### Validity and reliability

This measurement model consisted of five latent variables, including turnover intention, social support, mental health, stress overload and fatigue. Meanwhile, this measurement model was evaluated using the Confirmatory factor analysis (CFA) in the preliminary analyses. The CFA results indicated that there was an acceptable fit for the five factor: χ^2^ = 176.458, df = 35, *p* < 0 0.0001, RMSEA = 0.062, CFI = 0.983, TLI = 0.973, which demonstrated that it was a suitable fit to the data and this measurement model was acceptable. For each latent variable, the factor loading was significant, at 0.38–0.66. In addition, the Cronbach’s α values of each measure were also acceptable. Overall, above results illustrated that five latent variables were strong representatives of the latent constructs.

## Results

### Participants characteristics

There were 1100 theatre nurses taking part in this study, of which 1060 theatre nurses’ questionnaires were valid therefore the effective response rate was 96.36%. Of the 1060 theatre nurses investigated in this study, the median age was 31 (19, 59) years of age; 94.5% were junior college and undergraduate; 27.3% were unmarried and childless (n = 287), 66.5% were married and childbearing (n = 705), 6.4% were married and childless (n = 68). The participants mostly came from tertiary hospital (75.8%, n = 804), and they mostly worked in operating theatre less than 10 years (66.9%, n = 709). All results of the social-demographic and working characteristics are shown in Table 1.

**Table 1:** The social-demographic and working characteristics of participants (N = 1060)

Variables	Categories	Mean ± SD/N(%)
Gender	female	985(92.9)
	male	75(7.1)
Age(years)		32.94 ± 7.876
	≤ 25	193(18.2)
	26–30	295(27.8)
	31–35	246(23.2)
	36–40	147(13.9)
	> 40	179(16.9)
Education level	Technical secondary school	55(5.2)
	Junior college	543(51.2)
	Undergraduate and above	462(43.6)
Marital status	Single	289(27.3)
	Married	755(71.2)
	Divorced/Widowed	16(1.5)
Fertility status	Unmairried and childless	287(27.1)
	Married and childbearing	705(66.5)
	Married and childless	68(6.4)
Situation of raising children	Childless	287(27.1)
	Raising alonely	158(14.9)
	Raising with others	615(58)
Living status	Alone	125(11.8)
	With family	813(76.7)
	With friends	122(11.5)
Times of weekly exercise	0	510(48.1)
	1–2	450(42.5)
	≥ 3	100(9.4)
Chronic disease history	Yes	91(8.6)
	No	969(91.4)
Sufferring negative life events	Yes	196(18.5)
	No	864(81.5)
Hospital level	Tertiary hospitals	804(75.8)
	secondary hospital	256(24.2)
Hospital type	Special hospital	174(16.4)
	General hospital	886(83.6)
Employment status	Regular	451(42.5)
	Contract	609(57.5)
Professional title	Nurse	256(24.2)
	Secondary nurse	431(40.7)
	Chief nurse	309(29.2)
	Deputy chief nurse and above	64(6.0)
Working experiences (years)	≤ 5	309(29.2)
	5–10	311(29.3)
	10–15	159(15)
	15–20	104(9.8)
	> 20	177(16.7)
Working experience in OR(years)	≤ 5	397(37.5)
	5–10	312(29.4)
	10–15	149(14.1)
	15–20	73(6.9)
	> 20	129(12.2)
Weekly working hours(hours)	< 40	179(16.9)
	40–50	606(57.2)
	50–60	205(19.3)
	> 60	70(6.6)
Times of monthly night shifts	0	193(18.2)
	1–3	495(46.7)
	4–5	245(23.1)
	≥ 6	127(12)
Monthly income(yuan )	< 3000	97(9.2)
	3000–3999	177(16.7)
	4000–4999	231(21.8)
	≥ 5000	554(52.3)
Family support for jobs	support	795(75)
	a little bit support	248(23.4)
	nonsupport	3(0.3)
	encourage to quit the job	14(1.3)
Personal view for jobs	very like	201(19)
	like	391(36.9)
	a bit like	436(41.1)
	dislike	16(1.5)
	very dislike	16(15)
Why to choose nursing profession? (Multiple options)	Love of nursing	428(40.38)
	The need for survival	807(76.13)
	Meeting the wishes of parents	205(19.34)
	Other reasons	306(28.87)

### The comparison of turnover intention, social support, mental health, stress overload and fatigue scores

The score for turnover intention was 1.81 ± 0.86. The dimension of intention to leave profession had the highest score (2.09 ± 1.00), followed by intention to leave unit (1.86 ± 0.97) and intention to leave organization (1.72 ± 0.88). The score for mental health were 3.92 ± 2.36. The score of fatigue was 6.66 ± 3.60, physiological fatigue and psychological fatigue were 4.38 ± 2.77 and 2.28 ± 1.47, respectively. The score for social support was 65.38 ± 13.17, and family support was 22.05 ± 4.63, friend support was 21.6 ± 4.63, and other support was 21.73 ± 4.49. Further, the score of stress overload was 61.23 ± 16.44. All results are shown in Table 2.

**Table 2:** The comparision of social support, mental health, stress overload, fatigue and turnover intention scores (N = 1060)

Variables	Min	Max	Mean ± SD
Social support	12	84	65.38 ± 13.17
Family support	4	28	22.05 ± 4.63
Friend support	4	28	21.6 ± 4.63
Other support	4	28	21.73 ± 4.49
Mental health	0	11	3.92 ± 2.36
Stress Overload	22	110	61.23 ± 16.44
Event load	10	50	30.27 ± 7.67
Individual vulnerability	12	60	30.96 ± 9.74
Fatigue	0	14	6.66 ± 3.60
Physiological fatigue	0	8	4.38 ± 2.77
Psychological fatigue	0	6	2.28 ± 1.47
Turnover intention (Intention to leave)	1	5	1.81 ± 0.86
Intention to leave profession	1	5	2.09 ± 1.00
Intention to leave organization	1	5	1.72 ± 0.88
Intention to leave unit	1	5	1.86 ± 0.97

### The comparison of participant social-demographic, working characteristics and turnover intention

The single-factor analysis revealed that turnover intention among operating theatre nurses were significantly different in following facet: gender, age, situation raising children, times weekly exercise, chronic disease history, suffering negative life events, employment status, professional title, working experiences, working experience in operating theatre, weekly working hours, times of monthly night shifts, monthly income, family support for jobs and personal view for jobs (*p* < 0.05, Table 3).

**Table 3:** The comparison of the participant characteristics and the turnover intention (N = 1060)

Variables	Categories	Turnover intention	*t/*F Value	*P*
Gender	Male	2.01 ± 0.93	2.013	0.044*
	Female	1.80 ± 0.85		
Age(years)	≤ 25	1.84 ± 0.86	8.863	<0.001**
	26–30	1.92 ± 0.91		
	31–35	1.94 ± 0.90		
	36–40	1.75 ± 0.81		
	>40	1.50 ± 0.65		
Education level	Technical secondary school	1.77 ± 0.80	0.34	0.712
	Junior college	1.80 ± 0.87		
	Undergraduate and above	1.84 ± 0.85		
Marital status	Single	1.88 ± 0.87	1.598	0.203
	Married	1.79 ± 0.86		
	Divorced/Widowed	1.63 ± 0.74		
Fertility status	Unmarried and childless	1.92 ± 0.89	0.709	0.492
	Married and childless	1.77 ± 0.86		
	Married and childbearing	1.75 ± 0.80		
Situation of raising children	Childless	1.89 ± 0.87	3.527	0.03*
	Raising alone	1.66 ± 0.85		
	Raising with others	1.82 ± 0.85		
Living status	With family	1.79 ± 0.85	2.383	0.093
	With friends	1.97 ± 0.91		
	Alone	1.79 ± 0.81		
Times of weekly exercise	0	1.89 ± 0.88	4.496	0.011*
	2–3	1.77 ± 0.83		
	≥ 3	1.65 ± 0.84		
Chronic disease history	Yes	1.79 ± 0.85	2.962	0.003*
	No	2.07 ± 0.91		
Sufferring negative life events	Yes	1.77 ± 0.84	3.356	0.001*
	No	2.00 ± 0.92		
Hospital level	Tertiary hospitals	1.83 ± 0.88	1.216	0.225
	secondary hospital	1.76 ± 0.78		
Hospital type	Special hospital	1.75 ± 0.88	-1.154	0.249
	General hospital	1.83 ± 0.85		
Employment status	Regular	1.71 ± 0.84	-3.35	0.001*
	Contract	1.89 ± 0.86		
Professional title	Nurse	1.82 ± 0.86	4.013	0.007*
	Secondary nurse	1.89 ± 0.89		
	Chief nurse	1.78 ± 0.83		
	Deputy chief nurse and above	1.50 ± 0.67		
Working experiences (years)	≤ 5	1.85 ± 0.84	7.628	<0.001**
	>5 and <10	1.94 ± 0.93		
	>10 and ≤ 15	1.85 ± 0.89		
	>15 and ≤ 20	1.78 ± 0.80		
	>20	1.51 ± 0.67		
Working experience in operating theatre (years)	≤ 5	1.86 ± 0.87	5.175	<0.001**
	>5 and <10	1.90 ± 0.90		
	>10 and ≤ 15	1.82 ± 0.87		
	>15 and ≤ 20	1.66 ± 0.72		
	>20	1.53 ± 0.69		
Weekly working hours(hours)	<40	1.70 ± 0.81	5.972	<0.001**
	≥ 40 and ≤ 50	1.79 ± 0.84		
	>50 and ≤ 60	1.86 ± 0.88		
	>60	2.19 ± 0.99		
Times of monthly night shifts	0	1.63 ± 0.77	4.052	0.007**
	≥ 1 and ≤ 3	1.84 ± 0.87		
	≥ 4 and ≤ 5	1.88 ± 0.88		
	≥ 6	1.89 ± 0.88		
Monthly income(yuan )	<3000	1.96 ± 0.90	6.267	<0.001**
	3000–3999	1.95 ± 0.88		
	4000–4999	1.91 ± 0.95		
	≥ 5000	1.71 ± 0.78		
Family support for jobs	Support	1.68 ± 0.79	35.868	<0.001**
	A little bit support	2.18 ± 0.87		
	Nonsupport	2.90 ± 0.22		
	Encourage to quit the job	3.01 ± 1.11		
Personal view for jobs	Very like	1.40 ± 0.74	53.135	<0.001**
	Like	1.64 ± 0.72		
	A bit like	2.07 ± 0.85		
	Dislike	3.16 ± 0.58		
	Very dislike	3.08 ± 1.09		

**Annotation**: * means *p* < 0.05; * * means *p* < 0.01

### Correlation analysis of turnover intention, social support, mental health, stress overload, fatigue and their dimensions scores among operating theatre nurses

Correlation analyses showed that the turnover intention was negatively correlated with social support and its dimensions (*p* < 0.05), while it was positively related with mental health, stress overload, fatigue and their dimensions (*p* < 0.05, Table 4). Social support was negatively associated with turnover intention and its dimensions (*p* < 0.05), while it was negatively correlated with mental health, stress overload, fatigue and their dimensions (*p* < 0.05, Table 4). The mental health was positively correlated with stress overload, fatigue and their dimensions (*p* < 0.05, Table 4). Stress overload was positively related with the fatigue and its dimensions (*p* < 0.05, Table 4).

**Table 4:** Spearman correlation coefficients of Turnover intention, Social support, Mental health, Stress Overload, Fatigue and their factors score (r, N = 1060)

Variables	Turnover intention	ITLunit	ITLorg	ITLpro	Social support	Family support	Friend support	Other support	Mental health	Stress Overload	Event load	Individual vulnerability	Fatigue	Physiological fatigue	Psychological fatigue
Turnover intention	1														
ITLpro	0.805**	1													
ITLorg	0.971**	0.711**	1												
ITLunit	0.922**	0.688**	0.828**	1											
Social support	-0.266**	-0.248**	-0.262**	-0.221**	1										
Family support	-0.246**	-0.222**	-0.250**	-0.193**	0.945**	1									
Friend support	-0.255**	-0.240**	-0.248**	-0.218**	0.962**	0.850**	1								
Other support	-0.264**	-0.252**	-0.255**	-0.225**	0.967**	0.864**	0.916**	1							
Mental health	0.407**	0.384**	0.382**	0.369**	-0.381**	-0.375**	-0.361**	-0.360**	1						
Stress Overload	0.439**	0.437**	0.408**	0.395**	-0.209**	-0.203**	-0.204**	-0.192**	0.583**	1					
Event load	0.335**	0.360**	0.302**	0.306**	-0100**	-0.096**	-0.107**	-0.084**	0.452**	0.929**	1				
Individual vulnerability	0.477**	0.454**	0.451**	0.426**	-0.273**	-0.266**	-0.260**	-0.258**	0.629**	0.956**	0.780**	1			
Fatigue	0.298**	0.326**	0.263**	0.278**	-0.278**	-0.265**	-0.263**	-0.271**	0.480**	0.517**	0.467**	0.505**	1		
Physiological fatigue	0.254**	0.291**	0.218**	0.241**	-0.237**	-0.222**	-0.225**	-0.233**	0.426**	0.489**	0.458**	0.464**	0.925**	1	
Psychological fatigue	0.251**	0.248**	0.234**	0.226**	-0.234**	-0.228**	-0.220**	-0.225**	0.370**	0.344**	0.279**	0.361**	0.702**	0.380**	1

**Abbreviation**: (1) ITLunit: the scores of Intention to leave unit; (2) ITLorg: the scores of Intention to leave organization; (3) ITLpro: the scores of Intention to leave profession. *p < 0.05, ** *p* < 0.01

### Factors influencing turnover intention among operating theatre nurses

To find the factors influencing turnover intention among operating theatre nurses, all variables were entered and analyzed in the multiple linear regression, including the social-demographic and working characteristics, social support, mental health, stress overload and fatigue of operating theatre nurses. The results showed that there was no multicollinearity for all independent variables for turnover intention according collinearity diagnosis (Table 5). After adjusted analysis, eleven factors significantly influenced turnover intention among operating theatre nurses, including hospital level, gender, professional title, working experience in operating theatre, times of monthly night shifts, monthly income, personal view for jobs, family support for jobs, social support, mental health and stress overload (Table 5). These factors explained 32.2% of the total variance of turnover intention (F = 20.918, *p* < 0 0.05). However, fatigue had not significant influence on turnover intention.

**Table 5:** Multiple linear regression analysis for the factors of turnover intention (N = 1060)

Variables	B	SE	Beta	t	*p* ^†^	VIF	Adjusted R^2^	F
Turnover intention (Intention to leave)								
Hospital level	-0.134	0.059	-0.067	-2.283	0.023	1.343	0.322	20.918
Gender	-0.179	0.086	-0.054	-2.082	0.038	1.038		
Professional title	0.085	0.039	0.085	2.175	0.03	2.4		
Working experience in operating theatre	-0.091	0.025	-0.143	-3.708	< 0.001	2.328		
Times of monthly night shifts	0.062	0.025	0.065	2.426	0.015	1.116		
Monthly income	-0.086	0.025	-0.102	-3.431	0.001	1.372		
Personal view for jobs	0.181	0.031	0.178	5.758	< 0.001	1.485		
Family support for jobs	0.179	0.046	0.112	3.895	< 0.001	1.289		
Social support	-0.005	0.002	-0.073	-2.595	0.01	1.25		
Mental health	0.051	0.012	0.141	4.26	< 0.001	1.72		
Stress overload	0.013	0.002	0.256	7.959	< 0.001	1.62		

**Abbreviation**: B: standardized beta; SE: standard error; VIF: variance inflation factor. ^†^Adjusted for all other variables

### Structural equation modeling results

**Fig. 2:**
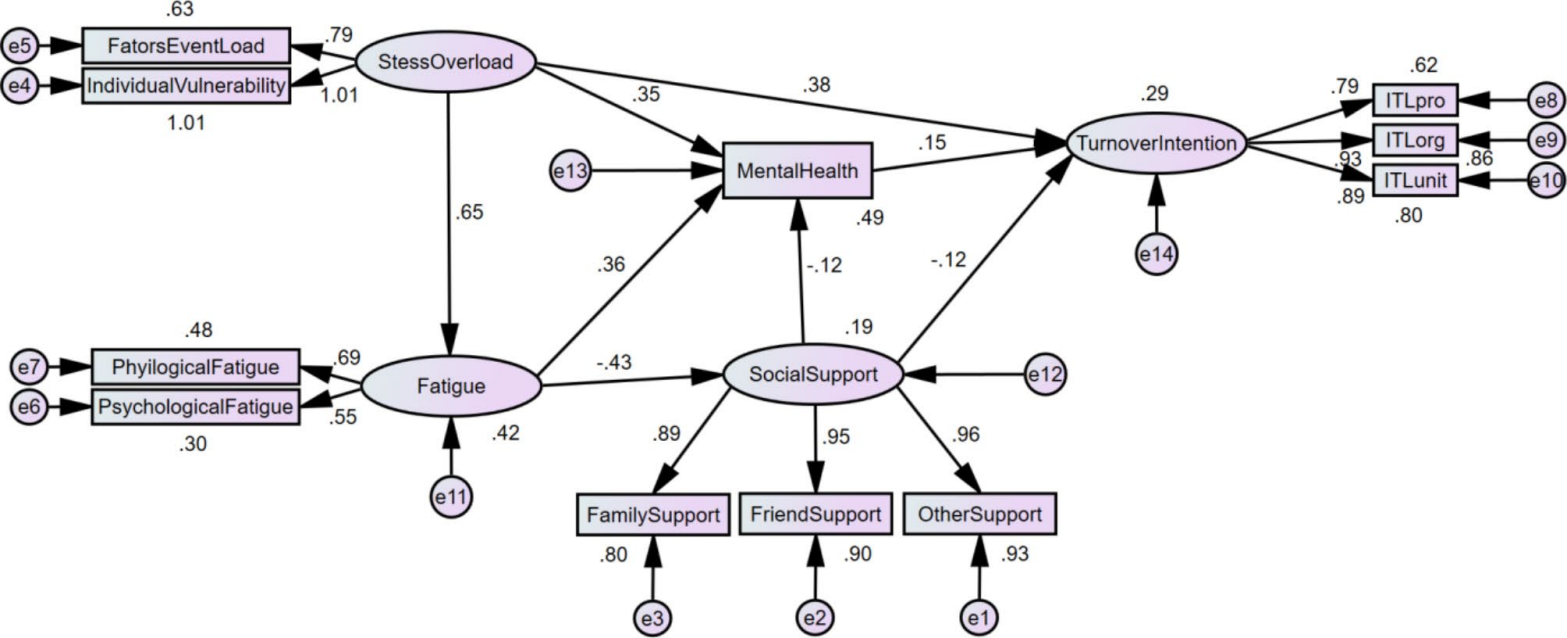
Mediating effects model of social support, mental health between stress overload, fatigue and turnover intention among operating theatre nurses

Based on the regression analysis, a structural equation model was further performed and tested the effects of social support, mental health, stress overload and fatigue on turnover intention among operating theatre nurses(Fig. 2; Table 6). In this model, the factor loading of respective observed variables and latent variables were 0.29 to 1.01; the path coefficients ranged from − 0.43 to 0.65 (Fig. 2), and all path coefficients had significantly statistics (*p* < 0.05, Table 6). The fitting indexes including χ^2^/df = 4.208, RMSEA = 0.055, GFI = 0.973, AGFI = 0.952, IFI = 0.918 and CFI = 0.917, were regarded as meeting the recommended criteria.

**Table 6:** The total effects, direct effects and indirect effects of each path in this model

Estimate	β	BC 95% CI^‡^		*p*
		Lower	Upper	
Total effects				
Stress overload→Mental health	0.619	0.581	0.657	< 0.001
Stress overload→Turnover intention	0.508	0.450	0.563	< 0.001
Fatigue→Mental health	0.414	0.318	0.522	< 0.001
Social support→Turnover intention	−0.139	−0.209	−0.071	< 0.001
Mental health→Turnover intention	0.150	0.079	0.223	< 0.001
Direct effects				
Stress overload→Fatigue	0.647	0.574	0.723	< 0.001
Stress overload→Mental health	0.351	0.260	0.429	< 0.001
Stress overload→Turnover intention	0.381	0.297	0.460	< 0.001
Fatigue→Social support	−0.434	−0.511	−0.359	< 0.001
Fatigue→Mental health	0.361	0.251	0.486	< 0.001
Social support→Mental health	−0.121	−0.183	−0.052	0.001
Social support→Turnover intention	−0.121	−0.191	−0.053	0.001
Mental health→Turnover intention	0.150	0.079	0.223	< 0.001
Indirect effects				
Stress overload→Fatigue→Social support	−0.281	−0.344	−0.266	< 0.001
Stress overload→Fatigue→Mental health	0.268	0.197	0.355	< 0.001
Stress overload→Fatigue→Social support→Turnover intention	0.127	0.083	0.175	< 0.001
Fatigue→Social support→Mental health	0.053	0.024	0.078	0.001
Fatigue→Social support→Mental health→Turnover intention	0.114	0.074	0.166	< 0.001
Social support→Mental health→Turnover intention	−0.018	−0.032	−0.007	0.001

**Abbreviations**: ^‡^Means that 95% bias-corrected bootstrap confidence interval

The results showed that stress overload had a positively direct effect on fatigue (β = 0.647, 95% confidence interval [0.574, 0.723]), which was supported for Hypothesis 1. Hypothesis 2 and 3 were also supported in this model. Stress overload had a positively direct and total effects on mental health (0.351[0.260, 0.429]; 0.619[0.581, 0.657], respectively). Stress overload had a positively direct and total effects on turnover intention (0.381[0.297, 0.460]; 0.508[0.450, 0.563], respectively). In addition, stress overload had positively indirect effects on turnover intention via fatigue and social support (0.127[0.083, 0.175]). Furthermore, fatigue had a negatively direct effect on social support (-0.434[-0.511, -0.359]), which was supported for Hypothesis 4. However, fatigue had a positively direct and total effects on mental health (0.351[0.260, 0.429]; 0.414[0.318, 0.522], respectively), and fatigue also had positively indirect effects on mental health via social support (0.053[0.024, 0.078]). All were supported for Hypothesis 5 and 6. Besides, social support had a negatively direct effect on mental health (-0.121[-0.183, -0.052]), which was supported for Hypothesis 7. And Hypothesis 8 was also supported in this model. Social support also had a negatively direct and total effects on turnover intention (-0.121[-0.191,-0.053]; -0.139[-0.209, -0.071], respectively), and fatigue had negatively indirect effects on turnover intention via mental health (-0.018[-0.032, -0.007]). The effect values of each path in this model was shown in Table 6. Overall, the results showed the fitness of the hypothetical model fitted well. Moreover, social support, mental health mediated between stress overload, fatigue and turnover intention among operating theatre nurses (Table 6).

## Discussion

In the wake of consistent epidemic of COVID-19 and an increasing care demands, the question that how to attract and retain operating theatre nurses are high attention of the nursing managers and researchers. The turnover of operating theatre nurses must further increases the nurses shortage. Turnover intention has been proved as a good predictor of the actual turnover behaviour [38, 48, 49]. Our research provided unique insights for nursing managers in better understanding the complex issue of turnover intention among operating theatre nurses.

In this study, the score for turnover intention among operating theatre nurses was 1.81 ± 0.86, which was lower than those in Lee et al. [37]. Because most nurses have a contradictory psychology towards the nursing work. Although the nursing work is under great pressure, nurses are still willing to stay in the nursing post for seeking their work stability [50]. Meanwhile, compared with a nurse in other nursing care units, operating theatre nurse needed spend more time and energy to be specialized and qualified in this profession [11]. Contrary to our results, Chen found that the overall level of turnover intention among operating theatre nurses was high, with a moderate and high proportion accounting for 78.64% [14]. However, the scale of turnover intention in Chen was different with this study [14]. So more studies should be further conducted to exactly explore the turnover intention among operating theatre nurses. Moreover, the score of the intention to leave profession in this study was highest compared with those of the intention to leave organization and the intention to leave unit, that was similar to previous studies [7, 37]. This must be related with the reasons that operating theatre nurses were faced with high work intensity, high responsibility, high pressure, high risk and closed environment in the operating theatre, and there are also a variety of potential risk factors for occupational hazards among operating theatre nurses.

Meanwhile, the regression results indicated that eleven influencing factors for turnover intention among operating theatre nurses were related to individual factors, health-related factors ans social work environment. That included hospital level, gender, professional title, working experience in operating theatre, times of monthly night shifts, monthly income, personal view for jobs, family support for jobs, social support, mental health and stress overload. However, fatigue had not significant influence on turnover intention. Similarly, Chen showed that influencing factors including salary, sense of fairness, operating theatre work characteristics, age, promotion opportunities, management methods have important effects on turnover intention among operating theatre nurses [14]. Besides, these finding were also similar to previous studies [7, 23, 35, 37, 38, 51]. Overall, age, salary and operating theatre work characteristics were remarkable “influencing factors” of nurses turnover intention. Nursing managers could take feasible measures to deal with above influencing factors of nurses turnover intention, and try to decrease the occurrence of turnover behavior.

After offered potential factors of turnover intention among operating theatre nurses, a structural model of this study also provided evidence that the relationships among social support, mental health, stress overload, fatigue and turnover intention. The results indicated that social support, mental health, stress overload and fatigue were important predictors of turnover intention among operating theatre nurses. These results were similar to Kim & Kim [6], which proved that job stress, burnout, job satisfaction, emotional labour, resilience, work environment, organizational commitment and job embeddedness were statistically significant predictors of nurses turnover intention globally. Besides, this model also revealed that stress overload positively affected fatigue, mental health and turnover intention; fatigue negatively affected social support, however, fatigue positively affected mental health; social support negatively affected mental health and turnover intention; mental health positively affected turnover intention. Moreover, social support, mental health mediated between stress overload, fatigue and turnover intention among operating theatre nurses. Liu et al. similarly found that perceived organizational support served as a mediator between burnout, job satisfaction, workplace violence and turnover intention among Chinese nurse [30]. Zhang et al. also indicated that social support had an indirect effect on nurses turnover intention via job satisfaction [52]. However, there were few study showed that mental health served as a mediated effect on turnover intention among operating theatre nurses. All hypotheses in this study regarding the relationships among variables were supported.

This study found that stress overload had positively direct and indirect effects on turnover intention among operating theatre nurses. It was similar to Huangpu [23], which showed that stress overload was positively related with turnover intention among emergency department nurses. Zhang et al. also found that job stress had greater direct effects on nurses turnover intention than organizational commitment and job satisfaction [52]. In addition, burnout and job stress had positive effect sizes on predicting nurses turnover intention [6]. This must be due to the fact that operating theatre nurses have to be faced with irregular commuter time, high frequency night shift, high work intensity and long operation time [14]. That caused operating theatre nurses being in a long-term state of tension and stress overload, and made them physically unbearable, so they had high level of intention to leave profession [14]. Therefore, operating theatre managers should pay dynamic attention to and timely assess the level of stress overload among operating theatre nurses [52], and reduce their stress overload through reasonable arrangement of shifts, so as to reduce their turnover intention.

Fatigue is strongly associated with physical and psychiatric disorders, especially, under continuous fatigue is easy to lead to the occurrence of disease [53]. Nurses’ work-related fatigue could threaten nurse physical and psychiatric health [54]. Our results proved that fatigue could positively affect mental health among operating theatre nurses. However, fatigue had not significantly effects on turnover intention in this study. This must be due to the fact that fatigue is a main feature of the burnout syndrome [31]. And burnout is a negative psychological experience where individuals were faced with highly interpersonal relationship in their job duties and highly exposure to stressors in long-term without adequate organizational support [55], including emotional exhaustion, depersonalization and low personal achievement [56]. Studies indicated that burnout had significantly positive direct effects and indirect effects on nurses turnover intention [55, 57]. Back et al. also showed that mediating effects of burnout on some aspects of emotional labor and turnover intention among Korean clinical nurses [55]. Moreover, this negative results suggested that we should focus on however “burnout” influenced nurses turnover intention more than “fatigue”. So nurse managers should undertake preventive measures or take effective intervention strategies or provide a safe occupational environment for operating theatre nurses to reduce work-related fatigue, and then reduce their burnout, and finally prevent their turnover intention.

Nurses mental health was associated with nursing quality, patients’ safety and satisfaction [33, 34]. In line with Kwon et al. [34], our results showed that operating theatre nurses had more mental health symptoms, like depressive and anxiety, they would had stronger willingness to turnover intention. Similarly, an increased level of fear of COVID-19 among front-line nurses was correlated with decreased job satisfaction, and increased intention to leave organization and profession [16]. Because operating theatre nurses had high incidence of anxiety and depression when being faced with the high risk of exposure to COVID-19 patients [21]. Meanwhile, operating theatre work characteristics like high work intensity and high learning intensity offer various challenges and psychological stress for operating theatre nurses. Besides, Li et al. found that social support was negatively associated with depression and anxiety severity among operating theatre nurses [21]. Supporting nurses from practical and psychological aspects is vital to preserving their health in the short and long term [18]. Hence, nursing managers should support operating theatre nurses mental health by providing peer and social support, psychological counselling, psychotherapy, long-term recovery support needs and so on [16, 18].

Social support is the emotional experience of the individual feeling supported, respected and understood [58]. We found that social support is the main factor affecting turnover intention among operating theatre nurses, which were similar to Meng [35]. However, these results were contrary to Li et al. [5], which showed that social support was not correlated with turnover intention among newly graduated nurses, while social support from friends and others was negatively associated with possibility of quitting the present job. It suggested that nursing managers could consider how to improve social support among operating theatre nurses from family, department, friends and other aspects, and establish a reasonable incentive system to decrease their turnover intention.

High turnover rates further increase the nurses shortage and then it must be detrimental to the patient care quality [37]. Our results had carefully analyzed turnover intention among operating theatre nurses, which could further be generalized to nursing managers facing similar nursing issues. Operating theatre managers could take feasible measures and effective intervention for operating theatre nurses to reduce stress overload and work-related fatigue, and provide peer social support to improve their mental health and turnover intention, and then finally prevent the occurrence of turnover behavior.

### Limitations

There are several limitations to consider in this study. First, although there were 1060 operating theatre nurses being analyzed in our study, the sample size was not representative of operating theatre nurses in China. Second, even though we had performed a structural equation model to analyze variables, the cross-sectional design still could carry bias for results. More longitudinal researches are needed to be further conducted for determining causal relationships among related variables. Finally, even though we had included many influencing factors of turnover intention in our study, there were still many important factors which were not included.

## Conclusion

To our knowledge, this is the first study to examine the hypothetical model for estimating the effects of stress overload and fatigue between social support, mental health and turnover intention among operating theatre nurses. Despite the limitations, our study also presented several important factors related to turnover intention among operating theatre nurses. This structural model indicated that social support, mental health, stress overload and fatigue were important predictors of turnover intention among operating theatre nurses. Moreover, social support, mental health mediated between stress overload, fatigue and turnover intention among operating theatre nurses. Overall, this study provided unique insights for nursing managers in better understanding the complex issue of turnover intention among operating theatre nurses. Nursing managers could take timely intervention measures to deal with the factors of turnover intention among operating theatre nurses, and try to decrease the occurrence of turnover behavior.

## Data Availability

All data are used and analyzed during the current study are available from the corresponding author on request.
